# Rural-Urban Disparity of Under-Five Mortality Rate in Iran from 1990 to 2015

**Published:** 2020-04

**Authors:** Younes MOHAMMADI, Manoochehr KARAMI, Nasrin DERAKHSHANZADEH

**Affiliations:** 1. Department of Epidemiology, School of Public Health, Hamadan University of Medical Sciences, Hamadan, Iran; 2. Social Determinants of Health Research Center, School of Public Health, Hamadan University of Medical Sciences, Hamadan, Iran; 3. Modelling of Noncommunicable Diseases Research Center, Hamadan University of Medical Sciences, Hamadan, Iran; 4. Students Research Committee, Hamadan University of Medical Sciences, Hamadan, Iran

**Keywords:** Infant mortality/trend, Child mortality/trend, Rural health, Urban health, Iran

## Abstract

**Background:**

To estimate under-five mortality rate (U5MR), as one of the sustainable development goals, in rural and urban areas of Iran from 1990 to 2015.

**Methods:**

We used the data collected through two censuses and one Demographic and Health Surveys (DHS). We analyzed the Summary Birth History (SBH) data via 2 approaches including Maternal Age Cohort (MAC) and Maternal Age Period (MAP) methods, and then, Gaussian Process Regression (GPR) was used to combine the three trends and with 95% uncertainty. Finally, Ratio of U5MR in rural to urban was calculated.

**Results:**

At the national level and in urban areas, U5MR in 1990, 2000, 2010, and 2015 was 66, 34, 18, and 13 per 1000 live births, respectively. Corresponding values in rural areas in 1990, 2000, 2010, and 2015 was 129, 64, 31, and 21 per 1000 live births, respectively. Accordingly, the ratio of U5MR in rural to urban at the national level was 1.93, 1.86, 1.72 and 1.63 in the same years. At the sub-national level, U5MR in urban areas ranged from 11.2 per 1000 live births in Isfahan to 18.2 per 1000 live births in Hormuzagn. U5MR in rural areas ranged from 14.1 per 1000 live births in Isfahan to 29.5 per 1000 live births in Sistan and Baluchistan

**Conclusion:**

There is still a gap between rural and urban areas, although it has decreased during the 25 years of the study. To alleviate this gap, health system authorities are advised to plan appropriate actions using multi-sectoral capacities

## Introduction

Under-five mortality rate (U5MR) is a major indicator of health and socio-economic status incorporated into development goals such as Millennium Development Goals (MDG), Sustainable Development Goals (SDG) and Burden of Disease and Risk factors. This indicator is used to reveal health disparities between and within communities (1).

The latest report by the WHO shows that about six million children die before the age of five and reports a wide disparity between countries. Under-five mortality rate in Iran reached 14 per 1000 live births in 2015. However, the estimate is an average that cannot reveal disparities between different areas of Iran including rural and urban areas (2).

Knowledge of geographical disparity especially between rural and urban areas is a vital source for health policy-making and planning and resource allocation. Although prior studies have confirmed disparity between rural and urban areas, our knowledge of the trend of child mortality in rural and urban areas of Iran and the magnitude of disparity between two areas is limited. One major reason for the shortage of data during past years is the lack or deficiency of death registration system (DRS) in Iran (3). Despite the utilization of DRS in Iran since 1996, the system has deficiencies; thus, the pure rely on the data produced by the system for assessing U5MR and disparity can result in misunderstanding. Therefore, researchers need to use alternative valid and reliable methods for estimating child mortality rate. Complete Birth History (CBH) and Summary Birth History (SBH) methods are considered as two alternatives for DRS. These methods have a satisfactory level of validity and reliability and suggested by international researchers (4). Considering the SDG which calls for a reduction in U5MR (less than 25 per 1000 live births by 2030) (5), it is necessary for Iranian health policy-makers to know the magnitude and determinates of disparities in U5MR between rural and urban areas at both national and sub-national levels to reduce disparities between rural and urban areas and achieve SDG. In this study, we aimed to estimate child mortality in rural and urban areas of Iran at both national and provincial levels from 1990 to 2015 using summary birth history method, and then estimate the degree of disparity between them.

## Methods

### Data sources

First, we estimated child mortality rate in rural and urban areas using SBH method. SBH method usually has two questions that asked from the 15–49 yr women: how many children ever born (CEB)? How many children ever survive (CES)? Clearly, no data on time and age can be collected via using these questions (6).

In this study, we extracted SBH from censuses 1996, 2006, and 2011, and DHS 2000. However, before estimating the rate, we assessed the quality of data sources using two criteria: the magnitude of missing variables and sex ratio. When the degree of missing data was over 10% and sex ratio was out of the normal range (1 to 1.06), the data were considered as low-quality data. Our quality assessment, based on the two criteria, showed that census 2011 and DHS 2010 had a low quality. Moreover, to reach reliable results, we analyzed the data sources and found that the estimates produced by the two mentioned sources were less than the DRS, which indicate the low quality of data. Therefore, we included censuses 1996 and 2006 and DHS 2000 in the final database.

### Statistical analysis

To analyze SBH questions, we used models and methods updated by Rajaratnam, et al. including Maternal Age Cohort (MAC) and Maternal Age Period (MAP) (4). Both methods use mother’s age as a proxy for children’s age. However, the approaches used by the two methods are different. MAC produces seven estimates of CMR based on seven age groups of mothers (15–19, 20–24…45–49), while MAP estimates CMR for every year prior to the survey.

The model of MAC method is as follows:

$$\text{log}\;\text{it}\left({}_{5}90_{ijk}\right)=\beta_{0i}+U_{ij}+\beta_{1i}\log it\left(\frac{CD_{ijk}}{CEB_{ijk}}\right)\beta_{2i}CEB_{ijk}+\beta_{3i}\frac{P{\left(15-19\right)}_{jk}}{P{\left(20-24\right)}_{jk}}+\beta_{4i}\frac{P{\left(20-24\right)}_{jk}}{P{\left(25-29\right)}_{jk}}+{ɛ}_{ijk}$$

Where,

_5_*q*_0_: = under-five mortality rate
i =: 5 - year maternal age group ∈ {15–19, 20–24, ..., 45–49}
j =: country
*k* =: *year of survey*
P(...) =: parity (average CEB) for specified maternal age group
CD_i_ =: total dead children from maternal age group
CEB_i_ =: total children ever born from maternal age group

The model of MAP method is as follow:

$$\log it\left(5q0_{tjk}\right)=\beta_{t}^{0}+U_{tj}+\beta_{t}^{1}\log it\left(\frac{CD_{tjk}}{CEB_{tjk}}\right)+{ɛ}_{tjk}$$

Where

t =: index of calendar time ∈ [0, 24]
j =: country
k =: survey
CD_tjk_ =: total dead children in time bint

In the next step, we combined the two estimates produced by MAC and MAP. Accordingly, we used Loess method, that it is a non-parametric smoothing method used for non-linear trends. We set bandwidth parameter by 0.5 (7).

As we used three data sources, we had three trends. In the next step, we integrated the three trends into one trend with 95% uncertainty. In this step, we used Gaussian Process Regression (GPR). This method is a Bayesian technique, which uses prior distribution and one likelihood function for producing posterior distribution. Prior distribution was obtained by use of Spatiotemporal model. The model includes the spatial and temporal components and covariates such as years of schooling and wealth index. Through sampling the posterior distribution, we reached the final estimate with its lower and upper bands. The detailed explanations of the methods are presented elsewhere (8, 9). We estimated U5MR in rural and urban areas at national and subnational levels separately. To calculate disparity ratio, we divided U5MR in the rural areas by U5MR in urban areas. STATA and R (R-Stan package) software were used to estimate U5MR.

## Results

**Fig. 1:**
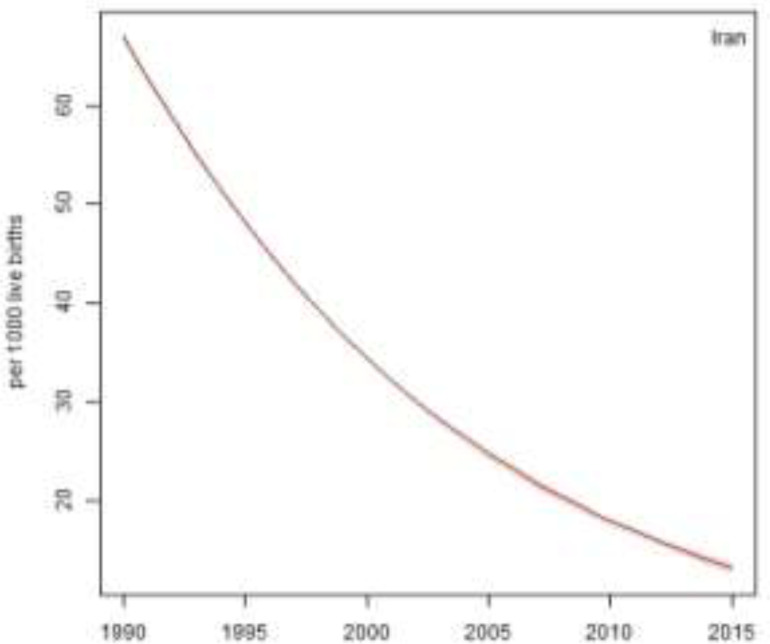
Under-five mortality rate in urban area of Iran from 1990 to 2015

The 25-year trend of under-five mortality rate in urban areas of Iran at the national level is presented in Fig. 1. U5MR in 1990 was 66.8 per 1000 live births, then it reduced to 34 per 1000 live births, 18 per 1000 live births, and 13 per 1000 live births in 2000, 2010, and 2015, respectively. Therefore, it indicates 80% reduction in U5MR from 1990 to 2015.

**Fig. 2:**
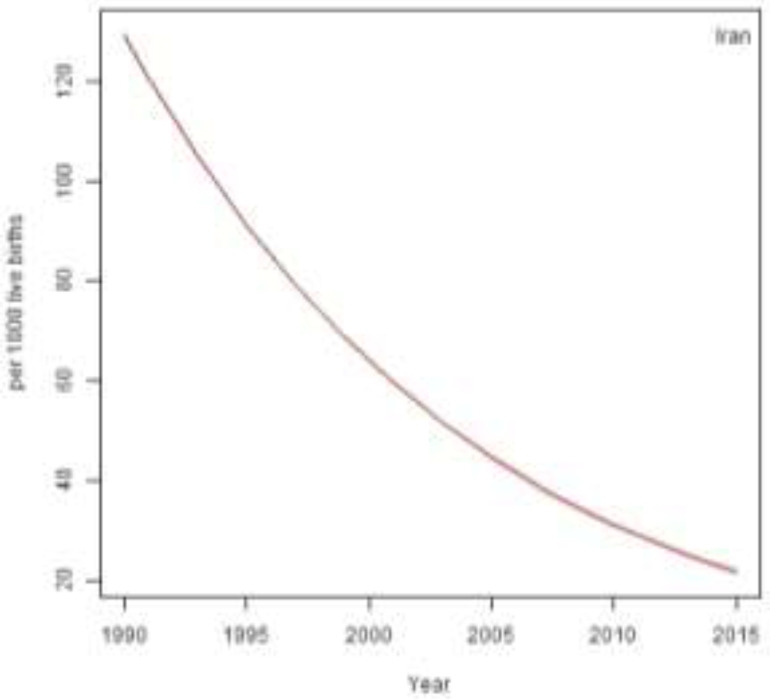
Under-five mortality rate in rural area of Iran from 1990 to 2015

Fig. 2 shows the 25-year trend of U5MR in rural areas of Iran from 1990 to 2015. This trend indicates 83% reduction in U5MR during 25 years. Accordingly, the estimated U5MR was 129 per 1000 live births in 1990 that reduced to 64 per 1000 live births in 2000, 31 per 1000 live births in 2010, and 21 per 1000 live births in 2015.

**Fig. 3:**
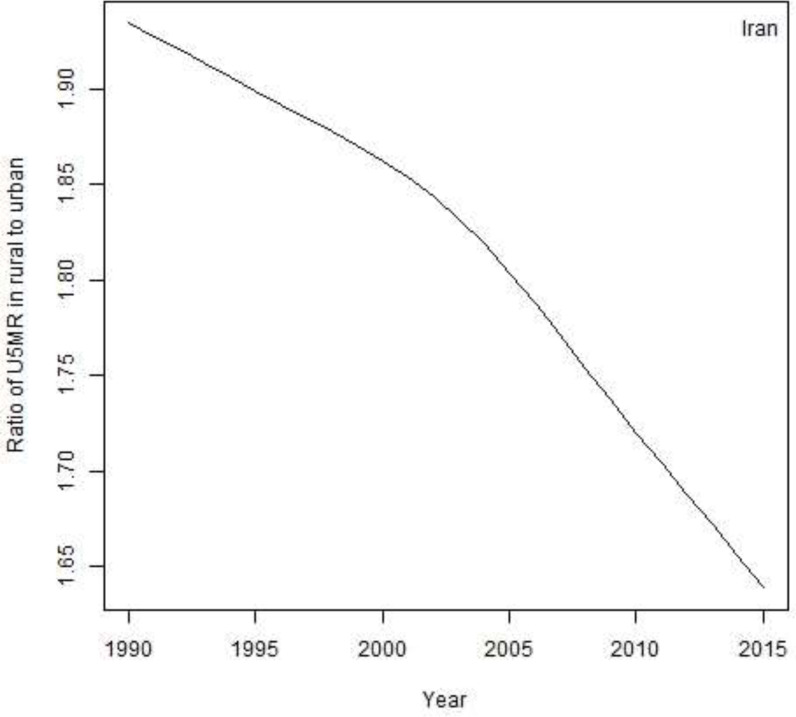
trend of ratio of U5MR in rural to urban

The trend of ratio of U5MR in rural to urban is presented in Fig. 3. The figure shows the decreasing trend of U5MR in rural areas, as it was 1.9 in 1990, then it reach to 1.8 in 2000 and 1.7 in 2010 and 1.6 in 2015.

U5MR at sub-national level had different patterns. The highest U5MR in urban areas in 2015 was observed in Hormuzgan Province (18 per 1000 live births), Sistan and Baluchistan Province (16 per 1000 live births), and Kurdistan and North Khorasan (15 per 1000 live births). On the other hand, Isfahan, Tehran, Qom, Mazandaran, Qazvin, and Markazi had the lowest U5MR (less than 12 per live births). The results for urban and rural areas are presented in Tables 1 and 2 respectively.

**Table 1:** The 25 years trend of under-five mortality rate in rural and urban areas of Iran at national and provincial levels

***Province***	***1990***		***2000***		***2010***		***2015***	
	***Urban***	***Rural***	***Urban***	***Rural***	***Urban***	***Rural***	***Urban***	***Rural***
Markazi	58.7(57.2–60.2)	104.3(101.7–107.2)	30.5(28.7–33)	52.9(49.8–57.2)	16.1(13.2–17.8)	25.3(20.8–28)	11.8(9–13.4)	17.2(13.1–19.5)
Gilan	65.3(64.1–66.4)	113.8(111.7–115.7)	32.7(32.3–33.7)	56.6(55.7–58.2)	16.9(16.4–17.7)	26.8(26–28.1)	12.3(11.4–13.3)	18.3(16.9–19.7)
Mazandaran	61.3(59.9–62.6)	95.4(93.3–97.5)	30.4(30–31.5)	45.9(45.4–47.6)	15.7(14.8–16.7)	22.7(21.4–24.1)	11.5(10.4–12.3)	16(14.5–17.2)
East Azerbaijan	66.4(65.1–68)	118(115.6–120.8)	33(32.5–34)	57.5(56.5–59.1)	16.8(15.7–18.3)	26.7(25–29.2)	12(10.6–13.9)	18.1(15.9–20.8)
West Azerbaijan	84(81.5–87.7)	154.4(149.7–161.1)	40.3(37.2–43.5)	72.9(67.4–78.9)	19.7(16.1–23.1)	34.8(28.3–40.7)	14(10.4–17)	24.2(17.9–29.5)
Kermanshah	79.2(77.8–80.6)	143.6(141.2–146.2)	39.9(38.9–41)	72.9(71.2–75)	20(19–20.8)	35.1(33.4–36.6)	14.2(13–15.1)	24(22–25.6)
Khuzestan	63.2(61.8–64.4)	116.3(113.8–118.6)	33.1(32.2–34.1)	59.4(57.8–61.1)	17.6(16.4–19.5)	29.5(27.5–32.7)	12.8(11.3–14.9)	20.5(18–23.8)
Fars	68.9(66.7–71.4)	135.2(130.8–140.2)	35.7(33.9–37.9)	68.9(65.5–73.1)	18.7(16–22.5)	34(29.1–41)	13.6(10.7–17.8)	23.7(18.7–31.2)
Kerman	73.6(72–75.1)	127.9(125.2–130.6)	37(36.7–38)	66.6(65.9–68.2)	19.3(18.7–20.2)	34(33–35.6)	14.1(13.3–15)	24(22.7–25.5)
Khorasan Razavi	67.7(65.3–68.9)	139.4(134.5–142)	35(34.2–36.2)	69.1(67.7–71.5)	18.4(17.1–19.7)	33.6(31.3–36.1)	13.4(11.9–14.5)	23.2(20.7–25.2)
Isfahan	55.3(53.8–56.5)	83.5(81.3–85.3)	29(24–30.8)	42.3(35–44.8)	15.4(7.7–18.3)	20.5(10.3–24.4)	11.2(4.3–14.4)	14.2(5.4–18.2)
Sistan and Baluchistan	83(81.9–84)	193.8(191.3–196.1)	43.2(42.7–44.2)	90.3(89.4–92.6)	22.1(20.2–25)	42.2(38.8–48)	15.7(13.6–19.3)	29.5(25.6–36.2)
Kurdistan	88.3(87.1–89.2)	161(158.7–162.6)	42.1(41.6–43.8)	77.9(76.8–80.9)	20.9(18.8–23.2)	37.2(33.4–41.3)	15(12.2–17.4)	25.5(20.8–29.6)
Hamadan	66.9(65.2–68.7)	114(111–116.9)	34.5(33–37.2)	57.4(54.9–61.9)	18.1(15.3–20.5)	27.3(23–30.9)	13.2(10.3–15.7)	18.5(14.5–21.9)
Chaharmahal and Balhtiari	62.7(61.5–64.3)	127.7(125.3–131)	31.8(30.9–34.1)	63(61.2–67.5)	16.5(15.3–18.2)	29.7(27.7–32.9)	11.9(10.5–13.3)	20.2(17.7–22.6)
Lorestan	71(69.9–72.3)	149.7(147.4–152.5)	34.1(33.2–34.9)	73.7(71.6–75.3)	16.8(15.9–17.8)	34.8(32.9–36.8)	12(10.6–13.1)	23.7(21.1–26)
Ilam	70.5(68.3–72.7)	132.2(128–136.2)	36.5(35.7–37.9)	65.9(64.4–68.5)	18.6(17.1–21.5)	31.5(29–36.5)	13.2(10.7–16.7)	21.6(17.5–27.3)
Kohgiluye and Buyerahmad	67.4(59.2–86.2)	136.9(120.3–175.1)	35.1(31–45.2)	64.5(56.9–83)	18.2(15.4–23.9)	28.7(24.4–37.6)	13.2(11–17.3)	18.8(15.7–24.8)
Bushehr	62.1(60.3–63.5)	108.2(104.9–110.6)	33.4(32.5–34.5)	54.4(53–56.2)	17.7(15.6–19.4)	26.5(23.4–29.1)	12.8(10.6–14.8)	18.5(15.4–21.4)
Zanjan	74.7(73.4–75.4)	156.8(154–158.3)	36.7(35.8–38.2)	71.1(69.6–74.2)	19.1(17.3–21.1)	33.5(30.4–37)	13.9(12–16)	22.9(19.7–26.3)
Semnan	61.4(60.7–62.1)	110.2(108.8–111.5)	32.1(31.6–32.9)	57.7(56.6–59)	17.1(16.3–18.3)	28.7(27.4–30.8)	12.5(11.3–13.8)	20.2(18.3–22.2)
Yazd	68.7(66.2–70.5)	106.1(102.3–109)	34.3(32.6–36.9)	55.7(53–59.9)	17.9(14.9–22.2)	28.2(23.6–35.1)	13.1(10.4–18)	19.8(15.7–27.2)
Hormuzgan	72.9(71.2–74.7)	133.3(130.1–136.6)	40.1(39.3–41.3)	69.8(68.4–71.9)	23.3(22.3–24.4)	37.2(35.6–39)	18.2(16.6–19.7)	27.7(25.3–29.9)
Tehran	52.4(50.5–53.3)	93.4(89.9–94.9)	27.9(26.6–30.2)	47.1(44.9–51.1)	15.3(13.2–18.3)	23(20–27.6)	11.4(8.5–16.9)	16.1(12–23.8)
Ardebil	73.2(71.3–74.4)	143.7(139.9–146.1)	35.3(33.9–37.4)	68.7(66.1–72.8)	17.8(14.9–20.9)	32.4(27.1–38)	12.8(9.7–16.5)	22.2(16.8–28.6)
Qom	55.8(54.4–57.1)	87.4(85.1–89.4)	28.9(28.4–30.2)	46.3(45.4–48.3)	15.5(14.6–17.3)	23.2(21.8–25.9)	11.5(10.4–13.1)	16(14.5–18.3)
Qazvin	59.5(58.1–61)	103(100.6–105.6)	30.3(29.7–31.2)	52.1(51.2–53.8)	15.9(14.9–17.4)	25.3(23.7–27.6)	11.7(10.4–13.4)	17.4(15.5–19.9)
Golestan	64.6(63.6–66.9)	126.2(124.2–130.7)	33.2(32.7–34.1)	63.3(62.3–65.1)	17.2(15.8–18.4)	30.8(28.3–32.9)	12.4(10.6–13.7)	21.3(18.1–23.5)
North Khorasan	75.1(73.3–77.4)	156.3(152.6–161.1)	38.4(37.5–40.2)	76.4(74.7–80)	20.2(19–21.4)	36.6(34.6–39)	14.7(12.4–16.9)	25.1(21.2–28.9)
South Khorasan	72.4(68.2–74.1)	167.2(157.5–171)	40(37.7–41.9)	81.7(77.2–85.9)	20.8(19.3–22.5)	39.7(36.8–42.9)	14.6(13.2–16.1)	27.5(24.9–30.4)
Alborz	58.7(57.2–60.1)	93.6(91.1–95.7)	30.6(29.3–31.9)	48.4(46.4–50.5)	16.3(13.5–20.8)	24.4(20.3–31.1)	12(8–19.1)	17.4(11.5–27.6)
National	66.8(66.2–67.3)	129.3(128.1–130.1)	34.4(34.3–34.9)	64(63.9–65)	18.1(17.7–18.5)	31.1(30.5–31.9)	13.2(12.7–13.7)	21.6(20.8–22.5)

**Table 2:** Ratio of under-five mortality rate in rural to urban by province from 1990 to 2015

***Province***	***1990***	***2000***	***2010***	***2015***
Markazi	1.8	1.7	1.6	1.5
Gilan	1.7	1.7	1.6	1.5
Mazandaran	1.6	1.5	1.4	1.4
East Azerbaijan	1.8	1.7	1.6	1.5
West Azerbaijan	1.8	1.8	1.8	1.7
Kermanshah	1.8	1.8	1.8	1.7
Khuzestan	1.8	1.8	1.7	1.6
Fars	2	1.9	1.8	1.7
Kerman	1.7	1.8	1.8	1.7
Khorasan Razavi	2.1	2	1.8	1.7
Isfahan	1.5	1.5	1.3	1.3
Sistan and Baluchestan	2.3	2.1	1.9	1.9
Kurdistan	1.8	1.9	1.8	1.7
Hamadan	1.7	1.7	1.5	1.4
Chaharmahal and Bakhtiari	2	2	1.8	1.7
Lorestan	2.1	2.2	2.1	2
Ilam	1.9	1.8	1.7	1.6
Kohgiluye and Buyerahmad	2	1.8	1.6	1.4
Bushehr	1.7	1.6	1.5	1.4
Zanjan	2.1	1.9	1.8	1.6
Semnan	1.8	1.8	1.7	1.6
Yazd	1.5	1.6	1.6	1.5
Hormuzgan	1.8	1.7	1.6	1.5
Tehran	1.8	1.7	1.5	1.4
Ardebil	2	1.9	1.8	1.7
Qom	1.6	1.6	1.5	1.4
Qazvin	1.7	1.7	1.6	1.5
Golestan	2	1.9	1.8	1.7
North Khorasan	2.1	2	1.8	1.7
South Khorasan	2.3	2	1.9	1.9
Alborz	1.6	1.6	1.5	1.4
National	1.9	1.9	1.7	1.6

Concerning ratio of U5MR in rural to urban, it ranged from 2 in Lorestan to 1.2 in Isfahan in 2015. The rest of provinces had the ratio between these two values.

## Discussion

This is the first paper estimating the trend of U5MR in rural and urban areas of Iran and assesses disparity between the two areas at both national and sub-national levels. Our findings at the national level showed a remarkable reduction (over 80%) in U5MR from 1990 (66.8 per 1000 live births) to 2015 (13.1 per 1000 live births) in urban areas of Iran. Moreover, there was 83% reduction in U5MR in rural areas as it was 129.2 per 1000 live births in 1990 and reached 21.6 per 1000 live births in 2015.

The results indicated the success of Iran in improving children survival rate in both rural and urban areas. This success is mainly due to the expansion of primary health care services in rural and urban areas since 1980’s. Given the need for easy and effective access to medical and health services for achieving Primary Health Care (PHC) goals, a Primary Health Care network was established for all parts of Iran in 1980 (10, 11). Accordingly, health houses and health centers are responsible for providing health and medical services in rural and urban areas, respectively. Health house is the smallest unit utilized for delivering health services in rural areas. Every health house has two personnel, so-called “Behvarz” (one male Behvarz and one female Behvarz), which are in charge of delivering health services (12). Each health house covers one main village and one or more sub-village. The services, delivered to children and mothers in health house, include anthropometry of infants, immunization program, family planning, health education, school health, environmental sanitation and health, occupational health, and screening for disease.

Examination of these indicators indicates the improvement of these activities over time. Due to the high coverage of immunization programs (over 95%) and environment sanitation, vaccine preventable diseases such as poliomyelitis, diarrheas, measles, mumps, and hepatitis, as the most important disease leading to mortality, are under the control or even eliminated in Iran (13, 14).

In addition to health and medical determinants affecting child mortality rate, non-health factors play a role in the reduction of child mortality rate (15). Like previous studies, we found a strong correlation the health and non-health factors and the difference between provinces and between rural and urban areas (16). Accordingly, investigating the results of other studies show that the provinces with a high U5MR had the lowest coverage of health services and vice-versa, and therefore, confirm the presence of a geographic disparity (17, 18). In addition, we found a strong correlation between U5MR and mother’s years of schooling and wealth index (coefficient correlation equal with 0.7). These two indices are non-health determinants of U5MR, reported by other studies as well (19, 20).

Although the rate of under-five mortality had improved in all provinces, we found a level of disparity between urban and rural areas over time. At national and sub-national levels, the magnitude of disparity had reduced. Our result shows a reduction of 15% during the 25 years of the study (from 1.93 in 1990 to 1.64 in 2015). Despite the reduction in disparity, the degree of disparity is continuous until 2015. The results of our study showed that the trend of both health and non-health related factors in rural areas had improved from 1990 to 2015. Furthermore, in provinces with high disparity between rural and urban areas in terms of U5MR, there was a gap in terms of health and non-health determinants. Inequality in the distribution of health and medical services might have been one of the major determinants of disparity between provinces and rural and urban areas. In addition, the family physician program, implemented in rural areas, is one of the important programs in Iran that increases people’s access to medical services for the treatment of diseases. The studies showed the positive effects of the program on health indicators in rural areas (21).

Due to the limitations of this study, it is necessary to be cautious when interpreting the results. First, we used summary birth history method, an indirect method, for estimating U5MR in rural and urban areas. Therefore, the results may not precisely depict the status of mortality. This method has assumptions such as lack of selection and information bias, that is, if the participants of the study differ from other mothers not participated in the study or do not enable to recall the birth events precisely the bias may ensue. As the second limitation, we did not use two recent surveys, DHS 2010 and census 2011. As we noted in the method section, we excluded them due to the low quality of data. Therefore, to estimate U5MR for recent years, we used statistical modeling based on covariates and the estimates for previous years; it might affect our results, especially for our findings for recent years, which it was reflected in confidence interval. The larger was the confidence interval, the more was uncertainty. Therefore, it is suggested for the researchers to use updated data sets for estimating U5MR in the next years.

## Conclusion

U5MR in both rural and urban areas of Iran have reduced substantially. However, there is a wide gap between urban and rural areas in Iran. To achieve SDG by 2030, health policy-makers of Iran should design and adopt measures for reduction disparities. It is suggested to improve the education level of mothers, create new jobs to promote wealth index, equally to distribute doctors, midwives, and Behvarz, and increase people’s access to water sanitation facilities.

## Ethical considerations

Ethical issues (Including plagiarism, informed consent, misconduct, data fabrication and/or falsification, double publication and/or submission, redundancy, etc.) have been completely observed by the authors.
